# 

**Published:** 1805-11-01

**Authors:** 


TO CORRESPONDENTS

Communications are received from Dr. Arneman, Dr. Patterson, Dr. Stanger, Mr. Barlow, Mr. Ring, and Mr. Simmons.

